# Draft genome sequences of two *Ralstonia mannitolilytica* strains (H3G44 and H3G46) isolated from the gut of captured *Tenualosa ilisha*

**DOI:** 10.1128/mra.00310-26

**Published:** 2026-05-18

**Authors:** Fahim Al Galib, Abdullah Al Kafi, Santu Biswas, Soharth Hasnat, Md. Shahrear Parvaj Sujon, Tahsin Islam Sakif, M. Nazmul Hoque, Morshedur Rahman, Md. Mahbubur Rahman, Jahangir Md. Alam, Dipali Rani Gupta, Tofazzal Islam

**Affiliations:** 1 Institute of Biotechnology and Genetic Engineering (IBGE), Gazipur Agricultural University (GAU) 198780, Gazipur, Bangladesh; 2 West Virginia University 5631, Morgantown, West Virginia, USA; 3 Department of Gynecology, Obstetrics and Reproductive Health, GAU, Gazipur, Bangladesh; 4 Department of Dairy and Poultry Science, Gazipur Agricultural University (GAU) 198780, Gazipur, Bangladesh; 5 Department of Biotechnology and Genetic Engineering, Islamic University 247297, Kushtia, Bangladesh; Montana State University, Bozeman, Montana, USA

**Keywords:** draft genome, Oxford Nanopore sequencing, symbiotic interactions, methylcitrate cycle, gut bacterium

## Abstract

We report draft genome sequences of *Ralstonia mannitolilytica* strains H3G44 and H3G46 isolated from the gut of captured *Tenualosa ilisha* in Bangladesh. Oxford Nanopore sequencing generated 4.76 and 4.77 Mb assemblies (66% GC) in three and two contigs, revealing metabolic traits linked to possible iron acquisition, stress response, and potential symbiotic interactions.

## ANNOUNCEMENT

The genus *Ralstonia* encompasses Gram-negative, oxidase-positive, non-fermentative rod-shaped bacteria commonly found in environmental settings such as water, soil, and plants (1, 2). The isolation of *Ralstonia mannitolilytica* from the gut of *Brahmina coriacea* raises curiosity about its role in the eukaryotic gut (3). In the current study, we purified two bacterial isolates, H3G44 and H3G46, from the gut of Bangladesh’s national fish, hilsa (*Tenualosa ilisha*), captured by local fishermen (gill net catch) from the Meghna River in Chandpur, Bangladesh (22.8333° N, 90.8333° E). Fish were processed immediately after transport on ice. The gastrointestinal tract was aseptically (sterilization with 70% ethanol) removed and homogenized in sterile PBS, then plated (serially diluted) on Brain Heart Infusion (BHI) agar (37°C for 24 h). Distinct colonies were purified by streaking, and H3G44 and H3G46 were cultured in BHI broth at 37°C for 24 h (180 rpm). Genomic DNA was extracted using the Tissue Genomic DNA Extraction HE Mini Kit (Favorgen, Taiwan) and quantified with Qubit 4.0 fluorometer (Thermo Fisher Scientific). Sequencing libraries were prepared using the Oxford Nanopore Technologies Ligation Sequencing Kit (SQK-NBD114.24) and the NEBNext Companion Module. No DNA shearing or size selection was performed. Libraries were barcoded with the Native Barcoding Kit 96 v14 and sequenced on the ONT MinION platform using R10.4.1 flow cells (4). Basecalling with Dorado v1.2.0 (SUP model) yielded 146,547 and 65,787 reads totaling 488,184,941 and 412,901,351 bp (N50: 6,656 and 11,877 bp). Quality assessment was performed with NanoPlot v1.46.1 (5); reads (>2 kb) were filtered using NanoFilt v2.8.0 (6); and *de novo* assembly was performed using Flye v2.9.5-b1801 (7). Assemblies were polished with four iterations of Racon v1.5.0 (8) and a final step with Medaka v2.0.1 (9); completeness and contamination were evaluated using CheckM v1.2.4 (10). The primary annotation present in the GenBank submission was generated via NCBI’s PGAP v6.10 (11), while specific metabolic features were identified using the RAST server (12), and plasmid replicons and CRISPR arrays were detected using PlasmidFinder v2.0.1 (13) and CRISPRCasFinder v2.0.3 (14), respectively. Default parameters were used for all software unless otherwise specified.

The 4.76 and 4.77 Mb draft genomes of strains H3G44 and H3G46 (66% GC) did not reveal major canonical virulence determinants under the annotation framework used here, and genome features are presented (Table 1). H3G44 and H3G46 were identified as *R. mannitolilytica* (ANI 99.78%–99.82%, >93% coverage) by GTDB-Tk v2.6.1 (15). Both genomes encode proteins associated with cell wall modification (e.g., NlpC/P60 family), biofilm formation (including the *pgaABCD* cluster in H3G44), and type IV pilus assembly (*pilZ *and *pilF*), which are commonly linked to surface attachment and environmental persistence. Previous studies have reported diverse bacterial species in the gut microbiome of hilsa (16, 17). Genes related to metabolic pathways such as the methylcitrate cycle and the protocatechuate branch were also identified, along with ABC-type Fe³^+^ transport systems. While *R. mannitolilytica* has been previously identified in the insect gut, here we report its presence within the fish gut microbiome. These predictive genomic features suggest a potential role in maintaining gut homeostasis and enhancing physiological resilience in hilsa. However, these inferred functional contributions require further experimental validation.

**TABLE 1:** Genomic features of two *R. mannitolilytica* strains (H3G44 and H3G46) isolated from the gut microbiota of *T. ilisha^a^*

Genome features	H3G44	H3G46
GenBank accession no.	JBSVSK000000000	JBSVSL000000000
SRA	SRR37535700	SRR37535699
Number of reads	146,547 (N50: 6,656)	65,787 (N50: 11,877)
Assembled genome size (bp)	4,759,758	4,771,849
GC content (%)	66	66
Coverage (×)	81	153
Genome completeness (%)	89.32% (CheckM)	99.52% (CheckM)
Contamination (%)	0%	0.34%
BioSample accession no.	SAMN53736892	SAMN53736893
N50 value (bp)	3,385,880	1,381,116
Number of contigs	3	2
Genes (total)	4,542	4,506
CDSs	4,480	4,444
Genes (coding)	4,076	4,374
CDSs (with protein)	4,076	4,374
Genes (RNA)	62	62
rRNAs	2, 2, 2 (5S, 16S, 23S)	2, 2, 2 (5S, 16S, 23S)
Complete rRNAs	2, 2, 2 (5S, 16S, 23S)	2, 2, 2 (5S, 16S, 23S)
tRNAs	52	52
ncRNAs	4	4
Pseudogenes	404	70
CDSs (without protein)	404	70
Pseudogenes (ambiguous residues)	0	0
Pseudogenes (frameshifted)	322	27
Pseudogenes (incomplete)	111	38
Pseudogenes (internal stop)	10	6
Pseudogenes (multiple problems)	38	11
CRISPR arrays	0	0
Plasmid replicon	0	0
Number of subsystems (metabolic features)	327	325

^*a*^ CDS, coding sequence.

## Data Availability

The whole genome shotgun project of the *R. mannitolilytica* strains (H3G44 and H3G46) has been deposited in GenBank under BioProject (Mining Bangladesh Biogold, accession number PRJNA1055760). The corresponding Sequence Read Archive accession numbers are SRR37535700 (H3G44) and SRR37535699 (H3G46). The versions described in this paper are versions JBSVSK000000000 and JBSVSL000000000.
